# Thalamic amplification of sensory input in experimental diabetes

**DOI:** 10.1111/ejn.13267

**Published:** 2016-05-30

**Authors:** Oliver J. Freeman, Mathew H. Evans, Garth J. S. Cooper, Rasmus S. Petersen, Natalie J. Gardiner

**Affiliations:** ^1^ Faculty of Life SciencesUniversity of ManchesterOxford RoadManchesterM13 9PTUK; ^2^ Centre for Advanced Discovery and Experimental Therapeutics (CADET)Central Manchester University Hospitals NHS Foundation TrustManchester Academic Health Sciences CentreManchesterUK; ^3^ Centre for Endocrinology and DiabetesInstitute of Human DevelopmentFaculty of Medical and Human SciencesUniversity of ManchesterManchesterUK; ^4^ School of Biological SciencesUniversity of AucklandAucklandNew Zealand; ^5^ Medical Sciences DivisionDepartment of PharmacologyUniversity of OxfordOxfordUK

**Keywords:** neuropathy, rat, somatosensory, streptozotocin, thalamus

## Abstract

Diabetic neuropathy is a common, and often debilitating, secondary complication of diabetes mellitus. As pain, hypersensitivity and paraesthesias present in a distal–proximal distribution, symptoms are generally believed to originate from damaged afferents within the peripheral nervous system. Increasing evidence suggests altered processing within the central nervous system in diabetic neuropathy contributes towards somatosensory dysfunction, but whether the accurate coding and relay of peripherally encoded information through the central nervous system is altered in diabetes is not understood. Here, we applied the strengths of the rodent whisker–barrel system to study primary afferent‐thalamic processing in diabetic neuropathy. We found that neurons in the thalamic ventral posteromedial nucleus from rats with experimental diabetic neuropathy showed increased firing to precisely graded, multidirectional whisker deflection compared to non‐diabetic rats. This thalamic hyperactivity occurred without any overt primary afferent dysfunction, as recordings from the trigeminal ganglion showed these primary afferents to be unaffected by diabetes. These findings suggest that central amplification can substantially transform ascending sensory input in diabetes, even in the absence of a barrage of ectopic primary afferent activity.

## Introduction

The somatosensory symptoms of diabetic neuropathy (DN) range from hypersensitivity, pain and paraesthesia to loss of sensation in 30–50% of patients with diabetes mellitus (Callaghan *et al*., 2012). In DN, symptoms typically first present in the distal limbs (Callaghan *et al*., 2012) and are generally believed to originate from ectopic firing of damaged primary afferents (Truini *et al*., 2013). Preclinical studies have found increased spontaneous activity, higher evoked firing rates and reduced ‘conduction failure’ in C‐fibres in peripheral nerves of streptozotocin (STZ)‐induced (Ahlgren *et al*., 1992; Sun *et al*., 2012) and BB/Wistar (Burchiel *et al*., 1985) diabetic rats. STZ‐diabetic rats also show increased spontaneous activity and lower activation thresholds in Aδ and Aβ fibres (Khan *et al*., 2002). Clinically, microneurography has revealed increased spontaneous firing in C‐fibres of patients with DN compared to healthy controls (Serra *et al*., 2012) and a higher percentage of unresponsive C‐fibres in patients (Ørstavik *et al*., 2006).

There is considerable evidence that facial nerves are also subject to DN. For example, in both clinical and experimental DN, there is loss of nerve fibres in the cornea, which originate from the ophthalmic division of the trigeminal nerve (reviewed in Papanas & Ziegler, 2015), a high prevalence of orofacial pain/burning mouth complaints in patients with DN (Arap *et al*., 2010) and reduced orofacial thermal, but not mechanical, thresholds in STZ‐induced diabetic rats (Rodella *et al*., 2000; Nones *et al*., 2013).

Evidence is also building for a significant contribution of the central nervous system (CNS) to neuropathic symptoms (Fischer & Waxman, 2010). The thalamus is the critical central gateway that exerts global control over communication to and from the cerebral cortex, so its role in neuropathic symptoms could be decisive. Patients with DN have increased functional connectivity (Cauda *et al*., 2009), increased blood flow (Selvarajah *et al*., 2011) and altered metabolite profiles (Selvarajah *et al*., 2008; Sorensen *et al*., 2008) in the thalamus. Amplification of ascending sensory signals within the CNS may contribute towards generation and maintenance of hyperalgesia, allodynia and altered sensation (Fischer & Waxman, 2010; Woolf, 2011). Compared to the dorsal root ganglia‐spinothalamic circuitry, much less is known about the electrophysiology of trigeminal‐thalamic changes in DN.

In the ventral posterolateral (VPL) thalamic nucleus, STZ‐diabetic rats show increased spontaneous and evoked activity to mechanical stimulation of the hindpaw (Fischer *et al*., 2009). Exactly how precise spike timing is affected by diabetes is unknown. Understanding whether neurons can accurately encode stimuli through somatosensory pathways may be pivotal to understanding why some patients with DN develop hypersensitivity and/or paraesthesia while others do not.

The rodent mystacial vibrissae (‘whisker’) system has tactile sensitivity comparable to the fingertips of primates (Carvell & Simons, 1990) and a classical trisynaptic circuit for processing information. It is an ideal model to investigate how mechanical stimuli encoded by peripheral primary afferents are handled by the thalamus in diabetes because: (i) the arrangement of whiskers on the rodent snout is highly stereotyped; (ii) each whisker is mapped to a distinct and discrete ‘barreloid’ in the ventral posteromedial (VPM) thalamus and (iii) individual whiskers can be accurately moved using computer‐controlled actuators (Petersen *et al*., 2009). These advantages mean that the precisely controlled, repeated comparisons in peripheral‐central transmission of sensory information between experimental groups can be achieved.

To explore the effects of diabetes on multiple levels of somatosensory processing, we recorded from both the VPM thalamus and the primary afferents of the rat whisker system (the trigeminal ganglion; TG) in response to a wide range of peripheral whisker stimulation in the STZ model of diabetes and age‐matched non‐diabetic control rats. These data provide novel insight into the relay of somatosensory information in diabetes.

## Materials and methods

### Animals

All procedures were performed in accordance with the UK Animal (Scientific Procedures) Act 1986 and University of Manchester ethical policies. Diabetes was induced in adult male Sprague–Dawley rats (268 ± 2 g, mean ± SEM; Harlan Laboratories) by intraperitoneal injection of 55 mg/kg STZ (Sigma; in 0.9% NaCl, *n = *17, Freeman *et al*., 2016). Weight/age‐matched controls (*n = *15) were injected with saline. Hyperglycaemia (> 15 mmol/L) was confirmed 3 days post‐STZ using a strip‐operated reflectance photometer (MediSense OptimumPlus).

Eleven weeks following STZ injection, animals underwent mechanical sensitivity testing (control *n *=* *9, diabetic *n *=* *10), following a modified protocol based on (Brussee *et al*., 2008). Rats were acclimatised for ~30 min in an elevated, wire mesh bottomed chamber (Ugo Basile). Von Frey filaments (4–26 g, Ugo Basile) were manually applied three times each to the left and right hind paw and the presence/absence of a hindlimb withdrawal was recorded. The percentage paw withdrawal across six stimulations is presented as median ± interquartile range and each force is analysed by a Wilcoxon signed‐rank test followed by *post hoc* Holm's sequential Bonferroni correction (MATLAB).

### Electrophysiology

Twelve weeks post‐STZ or saline injection, electrophysiology was performed on all control and diabetic rats [regardless of mechanical threshold or nerve conduction velocity (NCV) deficit] as previously described (Bale & Petersen, 2009; Bale *et al*., 2015). Briefly, rats were anaesthetised by 1.5 g/kg urethane (intraperitoneal, 30% w/v in saline), placed in a stereotaxic frame and maintained at 37 °C with a homeothermic blanket system.

For VPM recordings, a craniotomy was made contralateral to whisker stimulation, the dura was reflected and a single shank, 32‐channel silicon probe (recording site diameter 15 μm, site spacing 50 μm) was inserted vertically into the brain at coordinates between 2.5 and 3 mm lateral, 3.0–3.5 mm posterior. Whisker‐responsive units in the thalamus were found at a depth of between 4.5 and 6 mm from the pial surface. For TG recordings, a craniotomy was made ipsilateral to whisker stimulation, the dura was reflected and a tungsten microelectrode was inserted vertically into the brain at coordinates between 1.8 and 2.4 mm lateral, 1.0–2.0 mm posterior using a linear piezoelectric motor. Whisker‐responsive units in the TG were found at a depth of 9.5–11.0 mm from the pial surface.

Extracellular signals were pre‐amplified, digitised (sampling frequency 24.4 kHz), band‐pass filtered (300–3000 Hz) and continuously stored to hard disk for off‐line analysis. At the end of each experiment, motor and sensory NCV were measured in the sciatic nerve as previously described (Ali *et al*., 2014).

### Whisker stimulation

A unit's principal whisker was identified by manual deflection of individual vibrissae. Whiskers were cut to a length of ~15 mm from the skin and the principal whisker was inserted into a custom‐built, multi‐directional piezoelectric stimulator, ~10 mm from the skin (Bale & Petersen, 2009; Storchi *et al*., 2012).

In each of eight directions, whiskers were deflected by 10 equally spaced amplitudes between 40 μm and 400 μm. Each amplitude/direction combination was repeated 25 times in a randomised sequence. Each deflection lasted 250 ms, with 250 ms rest before the next deflection. To avoid mechanical resonance of the stimulator, square wave signals were smoothed by convolution with a Gaussian function (standard deviation 1.6 ms). An LED‐phototransistor device was used to verify that the stimulator reproduced the desired stimuli (Storchi *et al*., 2012).

### Electrophysiological data analysis

Electrophysiological data were analysed predominantly using MATLAB. Spikes emitted by a single unit were identified and isolated by thresholding and clustering in the space of 3–5 principal components using a mixture model. Only clusters whose interspike interval histogram exhibited a refractory period were accepted as single units (Bale & Petersen, 2009).

To analyse whisker responses, we computed peristimulus time histograms (PSTHs) for each amplitude/direction combination (bin width 20 ms). For each unit, the mean firing rate to the onset of the stimulus was computed over 25 repeated trials per amplitude/direction combination. Firing rate was defined as the mean firing rate in a 100 ms time window following stimulus onset. Spontaneous activity was computed as the mean/median firing rate in the 100 ms preceding each stimulus onset and compared with a student's *t* test and Mann–Whitney *U* test. Bursts were detected using established criteria for thalamic bursting: ≥ 2 spikes within 4 ms, preceded by 100 ms silence (Reinagel *et al*., 1999). Burst firing rates are presented as median ± interquartile range and analysed by Mann–Whitney *U* test (GraphPad Prism).

We tested whether a given unit was responsive to whisker deflection by comparing the spontaneous activity to the stimulus‐responsive firing rate (Wilcoxon signed‐rank test followed by *post hoc* Bonferroni correction). Non‐responsive units were rejected and are not presented. For comparisons across units, the preferred direction of each unit was defined as that which evoked the highest mean firing rate for the largest amplitude and tuning functions were aligned such that each unit's preferred direction was nominally 0°.

To test whether there was a difference in the tuning of units from diabetic rats to stimulus amplitude compared to units from control rats, we quantified the relationship between firing rate and stimulus amplitude. We fitted a quadratic polynomial to the tuning curve of the units from control rats and the units from diabetic rats in each direction and an extra sum‐of‐squares *F*‐test was performed with Bonferroni correction to deduce whether the two fits were significantly different (GraphPad Prism).

## Results

### Diabetic rats show a neuropathic phenotype

**Figure 1:**
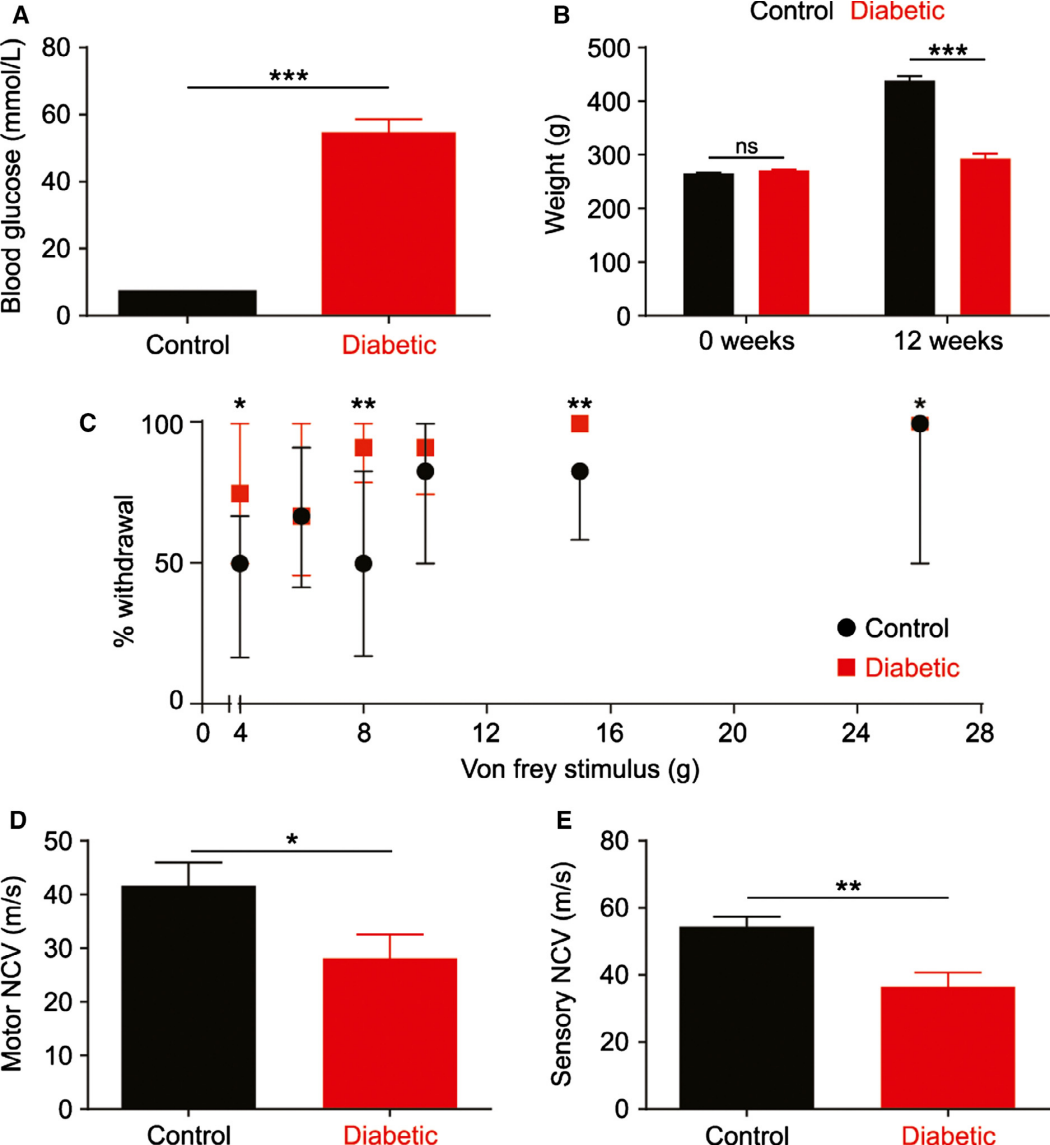
Diabetic rats show a neuropathic phenotype. Diabetic rats (red, *n *=* *17) were hyperglycaemic (A) and failed to gain weight (B) over 12 weeks of diabetes compared to non‐diabetic control rats (black, *n *=* *15). Diabetic rats showed hypersensitivity to manual Von Frey stimuli after 11 weeks (control *n *=* *9, diabetic *n *=* *10) (C) and reduced motor (D) and sensory (E) nerve conduction velocity (NCV) after 12 weeks (motor *n *=* *14, sensory *n *=* *13). Data are mean ± SEM and analysed by Student's *t* test (A,B,D,E) or median ± interquartile range and analysed by Wilcoxon signed‐rank test followed by *post hoc* Holm's sequential Bonferroni correction (C), *P <* *0.05, **P <* *0.01, ***P <* *0.001.

After STZ injection, rats developed long‐lasting hyperglycaemia (Fig. 1A, control *n *=* *15, diabetic *n *=* *17) and were lighter than age‐matched control rats by the end of the 12 week study (Fig. 1B). After 11 weeks of diabetes, rats were hypersensitive to mechanical stimulation of the hindpaw (Fig. 1C, control *n *=* *9, diabetic *n *=* *10). After 12 weeks, diabetic rats had significantly slower motor and sensory nerve conduction velocity (NCV) in the sciatic nerve compared to control rats (Fig. 1D: motor NCV, *n *=* *14; Fig. 1E: sensory NCV; *n *=* *13). These changes satisfy the criteria for a DN phenotype (Biessels *et al*., 2014).

### Thalamic hyperactivity to tactile stimuli in experimental diabetes

**Figure 2:**
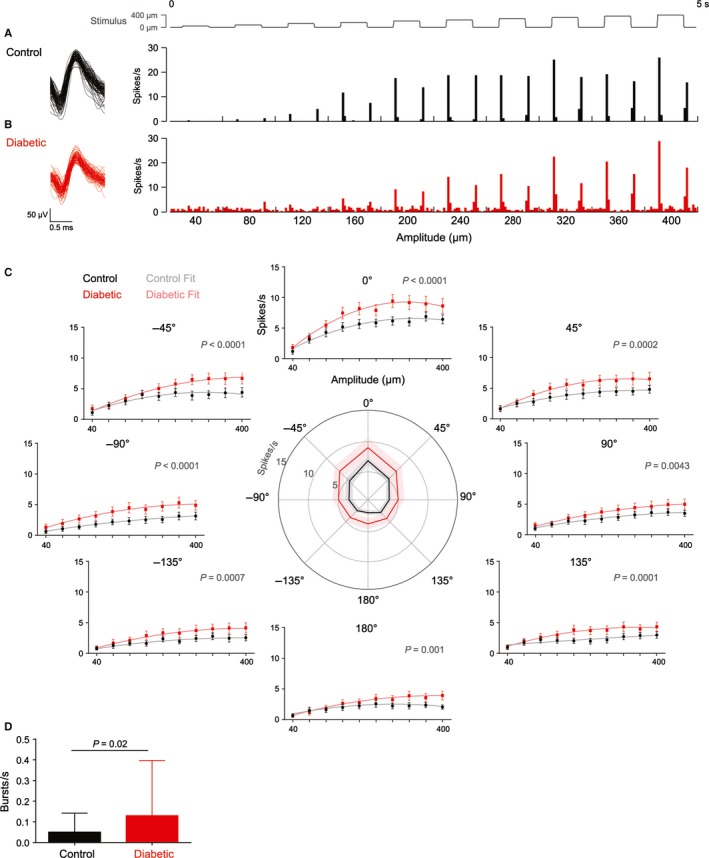
Thalamic hyperactivity in diabetes. Units were recorded from the ventral posteromedial (VPM) thalamic nucleus in response to graded whisker deflection in eight directions. Representative units from a control animal (A) and a diabetic animal (B) are shown. Quantitation of the firing rate to the onset of the stimulus (spikes/s) showed that on average, units from diabetic rats (red) fired significantly differently to units from control rats (black) across amplitudes in every direction (C). Plots show mean ± SEM and statistical comparison is between quadratic polynomial fits to the tuning curves of control (grey) and diabetic (light red) units, *P*‐value is shown in the top right of each plot. Central plot depicts the mean (solid line) ± SEM (shading) response to the largest amplitude (400 μm) in each of the eight directions (0 to ±180°). Control *n *=* *32 units from nine rats, diabetic *n *=* *37 units from 10 rats. Burst firing was higher in units from diabetic rats compared to controls (D), median ± interquartile range and analysed by Mann–Whitney *U* test.

We recorded a total of 32 well‐isolated single units from the VPM of control rats (*n *=* *9) and 37 from diabetic rats (*n *=* *10). To quantify the sensory selectivity of each unit, we delivered whisker deflection stimuli that varied in both amplitude and direction. In control rats, consistent with previous studies, we found firing rate increased with amplitude (Fig. 2A) and to peak in a neuron‐specific ‘preferred’ direction of deflection, designated 0° (Fig. 2C; Aguilar *et al*., 2008; Bale & Petersen, 2009). Although units from diabetic rats exhibited similar tuning (Fig. 2B), we found them, on average, to be significantly more responsive than units from control rats in every direction (Fig. 2C; all directions *P *<* *0.004). Thalamic relay neurons convey a substantial amount of information by firing in bursts, a firing mode which is thought to provide a powerful drive to cortex (Sherman, 2001). We found that thalamic units from diabetic rats fired significantly more bursts than those from control rats (control, 0.05 ± 0.12 bursts/s; diabetic, 0.13 ± 0.37; median ± interquartile range; *P *=* *0.02) (Fig. 2D), suggesting substantial amplification of sensory information occurs in diabetes.

### Thalamic hyperactivity is of central origin

What is the origin of these thalamic changes? As it is well‐established that diabetes causes changes in peripheral nerve function, we hypothesised that the thalamic hypersensitivity might simply be due to hypersensitivity in the trigeminal nerve. To investigate this possibility, we recorded from single primary afferents of the whisker system, whose cell bodies are located in the TG, under identical conditions to our VPM recordings.

**Figure 3:**
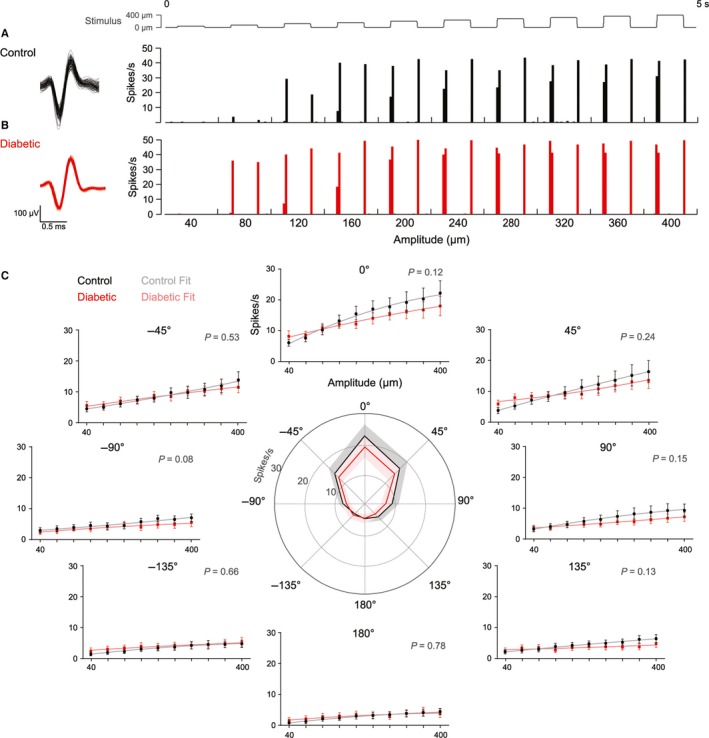
Preserved primary afferent activity. Units were recorded from the trigeminal ganglion (TG) in response to graded whisker deflection in eight directions. Representative units from a control animal (A) and a diabetic animal (B) are shown. Quantitation of the firing rate to the onset of the stimulus (spikes/s) showed that there was no difference in the firing behaviour of units from diabetic rats (red) compared to units from control rats (black) across amplitudes in every direction (C). Plots show mean ± SEM and statistical comparison is between quadratic polynomial fits to the tuning curves of control (grey) and diabetic (light red) units, *P*‐value is shown in the top right of each plot. Central plot depicts the mean (solid line) ± SEM (shading) response to the largest amplitude (400 μm) in each of the eight directions (0 to ±180°). Control *n *=* *31 units from six rats, diabetic *n *=* *25 units from seven rats.

We recorded a total of 31 well‐isolated, single units from the TG of control rats (*n *=* *6) and 25 from diabetic rats (*n *=* *7). In striking contrast to the VPM, TG responses to whisker deflection were unaffected by diabetes. Not only did units from diabetic rats display the tuning to amplitude and direction parameters typical of primary trigeminal afferents (Lichtenstein *et al*., 1990; Bale & Petersen, 2009) but, quantitatively, their responses to whisker deflection were statistically indistinguishable from those from control rats (Fig. 3). Taken together, these findings suggest a central origin for the thalamic hypersensitivity.

### Thalamic hyperactivity is both spontaneous and evoked

**Figure 4:**
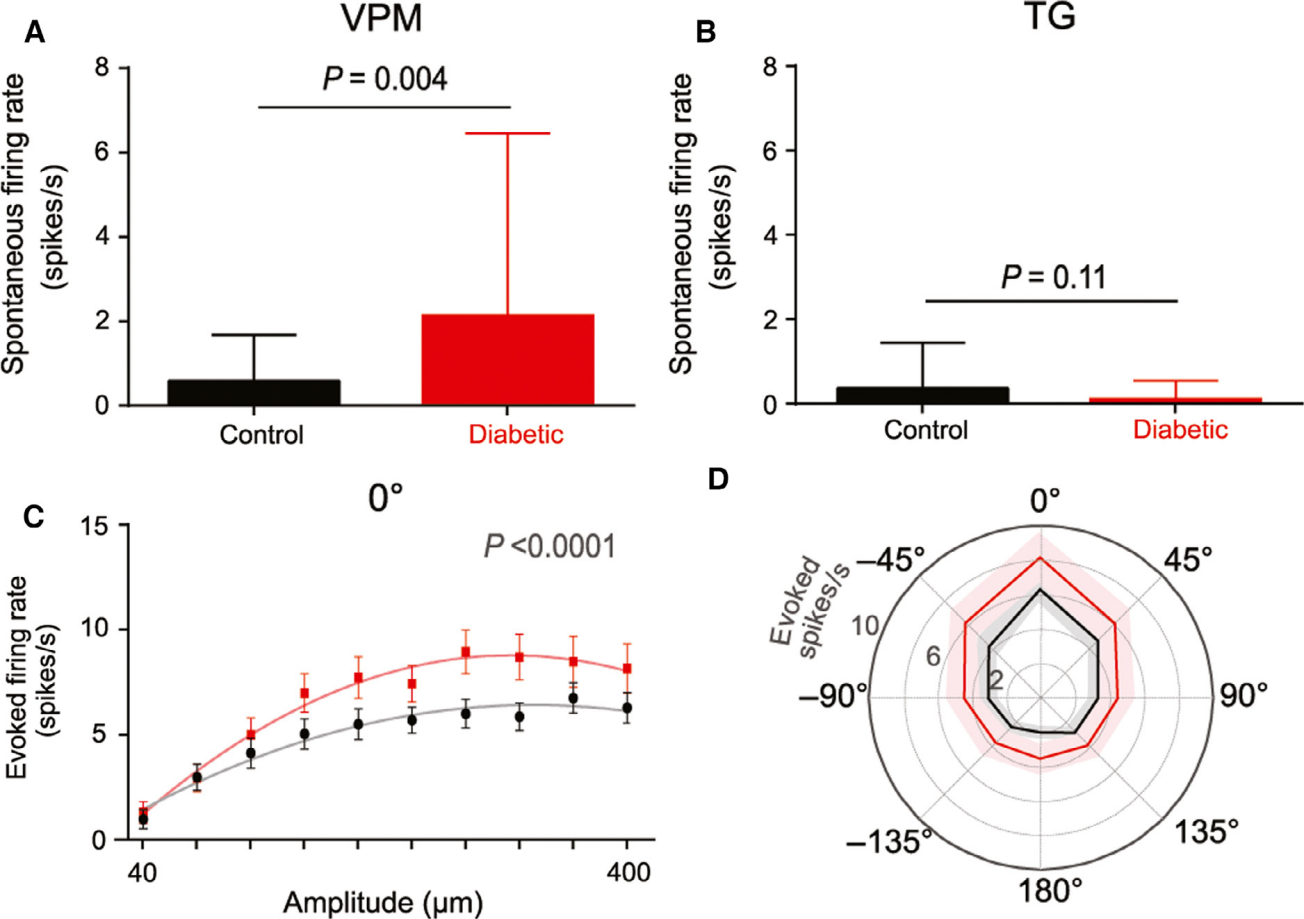
Thalamic hyperactivity is both spontaneous and evoked. There was a significantly greater spontaneous firing rate in units from the ventral posteromedial (VPM) thalamic nuclei of diabetic animals (*n *=* *37 units) compared to controls (*n *=* *32 units, median ± interquartile range, analysed by Mann–Whitney *U* test) (A). Spontaneous firing rate did not differ between control (black, *n *=* *31) and diabetic (red, *n *=* *25) units in the trigeminal ganglion (TG) (B). When spontaneous activity of VPM units was subtracted from their ‘raw’ firing rate (Fig. 2) to give the evoked firing rate, firing behaviour of units from diabetic rats remained significantly different to units from control rats across amplitudes in their preferred direction (0°) (C). Plot shows mean ± SEM and statistical comparison is between quadratic polynomial fits to the tuning curves of control (grey) and diabetic (light red) units. This was consistent across 7/8 directions (D), for 90° *P *=* *0.09, all other directions *P *<* *0.02. Mean (solid line) ± SEM (shading) response to the largest amplitude (400 μm).

In principle, thalamic hyperactivity could reflect a generalised increase in spontaneous firing and/or an increase in sensitivity to sensory input. To investigate, we compared spontaneous firing rates between diabetic and control rats. In the VPM, we found a significantly higher spontaneous firing rate in units from diabetic rats (2.1 ± 5.7 spikes/s; median ± interquartile range) compared to units from control rats (0.6 ± 1.5; *P *=* *0.004; mean ± SEM: control 5.2 ± 7.4, diabetic 1.8 ± 3.3; *P *=* *0.02; Fig. 4A). Consistent with previous studies (Lichtenstein *et al*., 1990; Bale & Petersen, 2009; Bale *et al*., 2013), we found spontaneous firing in the TG of units from control rats to be low/absent (0.3 ± 1.4 spikes/s; median ± interquartile range), which was also the case in units from diabetic rats (0.1 ± 0.5; *P *=* *0.11; mean ± SEM: control 1.3 ± 2.4, diabetic 0.4 ± 0.8; *P *=* *0.08; Fig. 4B).

To test whether the greater sensory responsiveness of thalamic units in diabetes might be explained by increased spontaneous thalamic activity, we subtracted each unit's spontaneous activity from the ‘raw’ firing rate (presented in Fig. 2) to give the ‘evoked’ firing rate. We found that the evoked firing rate remained significantly different between control and diabetic groups across amplitudes (at 0° *P *<* *0.0001; Fig. 4C) in 7/8 directions (for 90° *P *=* *0.09, all other directions *P *<* *0.02; Fig. 4D), suggesting that both spontaneous and evoked firing contribute to thalamic hypersensitivity in diabetes. Collectively, these results may indicate a multi‐faceted thalamic amplification of ascending peripheral input in diabetes.

## Discussion

The salient result of this study was that experimental diabetes leads to substantial hypersensitivity in the trigeminal thalamus and that this effect appears of central, not peripheral, origin. Our work adds to an emerging view that altered thalamic function may play a major role in the pathophysiology of DN (Selvarajah *et al*., 2008; Sorensen *et al*., 2008; Fischer *et al*., 2009; Fischer & Waxman, 2010). The amplification of ascending peripheral input could contribute towards the neuropathic symptoms of hypersensitivity, pain and/or paraesthesia (Woolf, 2011).

We show that in experimental diabetes, ascending input can be centrally amplified without overt dysfunction of trigeminal afferents. The mechanical responses of TG primary afferents were similar in diabetic and control rats and were consistent with previous reports (Lichtenstein *et al*., 1990; Bale & Petersen, 2009), suggesting central amplification of sensory information in the VPM was not simply due to increased firing of primary afferents. There are several possible mechanisms for this VPM hypersensitivity: peripheral deafferentation; a change in ascending input from the brainstem; and/or a change in central inhibition.

Deafferentation has been linked to thalamic reorganisation and repetitive bursting following nerve injury or lesion (Garraghty & Kaas, 1991a,b; Fitzek *et al*., 2001; Weng *et al*., 2003). There is evidence that trigeminal sensory nerves can degenerate in diabetes: loss of corneal nerve fibres has been reported in both clinical and experimental diabetic neuropathy (Papanas & Ziegler, 2015). However, in STZ‐induced diabetes, a significant decrease of corneal nerve fibres occurred only from 32 weeks post‐STZ (Chen *et al*., 2013) compared to the 12 weeks of this study. While we did not have difficulty finding vibrissae‐responsive neurons in the TG of diabetic animals compared to controls, we cannot rule out a potential contributory effect of distal deafferentation to thalamic hyperactivity.

It is known that the heightened spontaneous activity in the VPL in experimental diabetes persists after cessation of peripheral input, suggesting a central mechanism for the maintenance of hyperactivity (Fischer *et al*., 2009; Fischer & Waxman, 2010). Moreover, our results are consistent with imaging evidence for increased activity in both VPL and VPM after 6 weeks of STZ‐induced diabetes (Paulson *et al*., 2007). A second possible explanation for VPM hypersensitivity might be dysfunction of the brainstem trigeminal complex. Neurons in the principal (Pr5) and spinal (Sp5) trigeminal nuclei relay signals from the TG to the VPM and are analogous to the second‐order neurons of the dorsal horn of the spinal cord. Diabetes‐associated biochemical dysfunction has been described in STZ rats in the caudal division (Sp5C) that relays nociceptive information: STZ‐diabetic rats have increased protein kinase Cγ (Xie *et al*., 2015) and decreased glutamate net content and uptake in Sp5C compared to healthy control animals (Maneuf *et al*., 2004). However, changes in Pr5 have not been examined in diabetes. It will be important for future studies to determine at which stage of the ascending pathway amplification is first evident.

Finally, VPM hypersensitivity might occur directly in the thalamus due to loss of thalamic inhibition. While the rat VPM does not contain inhibitory interneurons, GABAergic innervation does arise from the thalamic reticular nucleus (NRT) (Barbaresi *et al*., 1986). Inactivation of the NRT by either electrolytic lesion (Lee *et al*., 1994) or pharmacological inhibition (Hartings & Simons, 2000) substantially increases spontaneous firing rate in VPM. Although it is not known whether NRT is affected in diabetes, there is evidence for GABAergic involvement in other neuropathies. Patients with painful trigeminal neuropathy show decreased thalamic grey matter volume, decreased thalamic GABA and decreased cerebral blood flow in the reticular nucleus (Henderson *et al*., 2013). In a peripheral nerve injury model of neuropathic pain, ~20% of GABAergic interneurons in the spinal dorsal horn are lost (Scholz *et al*., 2005). It would thus be interesting for future studies to assay GABA and its receptors in both the VPM and NRT in diabetes to determine whether loss of GABAergic signalling contributes to the increased VPM activity.

In summary, we found that the firing rate of whisker primary afferents were unaffected by STZ‐diabetes but that neurons within the VPM thalamus were hyperactive. With the knowledge that the VPL is autonomously hyperactive in experimental diabetes (Fischer *et al*., 2009), the current findings suggest that central amplification of sensation may occur without a barrage of aberrant primary afferent activity. Future research targeting the molecular basis of autonomous thalamic hyperactivity may yield exciting new therapeutic candidates to treat the debilitating symptoms of DN.

## Conflict of interest

The authors declare no competing interests.
